# Case report: Cerebellar sparing in juvenile Huntington's disease

**DOI:** 10.3389/fneur.2022.1089193

**Published:** 2023-01-11

**Authors:** Bruno Lopes Santos-Lobato, Jéssica Santos de Souza Rocha, Luciano Chaves Rocha

**Affiliations:** ^1^Laboratory of Experimental Neuropathology, Federal University of Pará, Belém, PA, Brazil; ^2^Hospital Ophir Loyola, Belém, PA, Brazil

**Keywords:** juvenile Huntington's disease, cerebellum, magnetic resonance imaging, atrophy, case report

## Abstract

Juvenile Huntington's disease is an early-onset variant of Huntington's disease, generally associated with large CAG repeats and distinct clinical symptoms. The role of the cerebellum in Huntington's disease has been reevaluated, based on the presence of ataxia and findings on the impact of the disease on cerebellar volume. Recent studies showed a hyperconnectivity between the cerebellum and the basal ganglia in premanifest children with expanded CAG repeats, as well as an enlargement of the cerebellum in adolescence-onset Huntington's disease. We report a 21-year-old Brazilian female with Huntington's disease (age at disease onset 16 years) with Parkinsonism and no ataxic features. There was no reduction of cerebellar volume over 3 years of follow-up, despite the brain atrophy in other regions and clinical worsening. Furthermore, the cerebellar volume of the patient was similar to age- and sex-matched controls. These findings support the existence of compensatory mechanisms involving the cerebellum in individuals with a moderate-to-high number of CAG repeats (50–100 copies) in the early stages of life.

## Introduction

Juvenile Huntington's disease (JHD) is a rare variant of Huntington's disease (HD) with age at onset before 21 years old. The prevalence of JHD occurs in 1.4% of all HD cases (1). It may present in two forms: (I) a childhood-onset HD, with the onset of symptoms usually before 10 years of age, characterized by a delay in developmental milestones, regression in cognitive skills, seizures, multiple motor features (ataxia, oropharyngeal dysfunction, dystonia), and with very large CAG repeats; (II) an adolescence-onset HD, with age at onset in the second decade of life, more associated with decline in academic performance, behavioral/psychiatric manifestations, oropharyngeal dysfunction, and Parkinsonism (bradykinesia, rigidity, rest tremor, and postural instability) (2). Chorea is not a common symptom in JHD as in adult-onset HD.

The role of the cerebellum in HD has been questioned recently, with evidence of mild cerebellar atrophy in adult-onset HD (3) and severe cerebellar atrophy in childhood-onset HD with extreme CAG repeats (4, 5). In adult-onset HD, cerebellar atrophy is associated with a higher motor disease burden and psychiatric abnormalities (6). Ataxia occurs in ~70% of patients with HD (7). However, there are few analyses of cerebellar volume in adolescence-onset HD using MRI volumetry or neuropathology. A recent study reported that patients with JHD with a mean age at disease onset between 10 and 20 years had an enlargement in cerebellar volume, compared with healthy controls, probably due to a compensatory mechanism (8). Supporting this hypothesis, we report an adolescence-onset HD patient with cerebellar sparing of brain atrophy over 3 years of follow-up through volumetric analysis.

## Case presentation

An 18-year-old woman was evaluated due to progressive rigidity and bradykinesia. She was the only daughter of a non-consanguineous couple with many affected family members with an incapacitant unknown disease with paternal inheritance, including her father. She presented developmental delay in school since early childhood and marked psychological and cognitive impairment at 16. At the same time, she developed rigidity and bradykinesia on her left leg. At 18 years of age, she was diagnosed with Parkinsonism, and levodopa was prescribed, with no improvement of motor symptoms. On physical examination, there was mild rigidity and moderate bradykinesia, slightly worse on her left side, with discrete and symmetrical rest tremor. Examination of extraocular movements showed hypometric saccades without nystagmus. Ataxic abnormalities in the finger-to-nose and heel-to-shin test, dysdiadochokinesia, and wide-based gait were not found. There was no previous description of chorea or seizures. She was submitted to a panel for genetic Parkinsonism, and the result was negative. MRI of the brain revealed mild caudate and putamen atrophy. At 20 years of age, genetic testing for HD demonstrated 56 triplet repeats on one allele and 20 on the other. At 21 years of age, a second MRI of the brain was performed, with a progression of global brain atrophy. More recently, the patient developed complex visual hallucinations after her father's death, and no choreic movements were described.

### Image acquisition and processing

MRI of the patient was performed on a 1.5-T unit (Philips Healthcare, Best, The Netherlands) using contiguous sagittal 3-D fluid-attenuated inversion recovery pulse sequence (TR 11.8 ms, TE 5.7 ms, acquisition matrix: 512 × 512, slice thickness: 1.2 mm). We performed MRI at two points: at 18 years (t1) and 21 years of age (t2). For comparison of brain volume between the patient and non-affected individuals, MRI data from normal age-matched female controls were acquired on a 1.5-T unit (Siemens Healthcare, Erlangen, Germany), using a high-resolution T1-weighted MPRAGE anatomical scan (TR 2.73 ms, TE 3.57 ms, slice thickness = 1.0 mm) as previously described (9). We selected eight female controls (two 18 years-old and six 19 years-old women—control group 1) for t1 matching and eleven female controls (five 20 years-old and six 21 years-old women—control group 2) for t2 matching.

For all DICOM files from the patient and controls, a preprocessing phase was performed using the freeware MRIcroGL (10): the aligned files were reconstructed by volume modeling and saved as a neuroimaging informatics technology initiative (NIFTI) file. After, we conducted a global automatic segmentation of the brain with vol2Brain, and a specific automatic segmentation of the cerebellum with CERES online volumetry softwares (IBIME, Valencia, Spain), as previously described (11, 12). The volume of brain regions for analysis with vol2Brain included summary data, neocortical gray matter areas, and subcortical areas. The volume of the cerebellum for analysis with CERES included total cerebellum and cerebellar lobules.

### Statistical analysis

Segmented structure volumes in cm^3^ of the patient were compared to the mean structure volumes of age-matched control groups 1 (matched with patient's t1) and 2 (matched with patient's t2) by using a *Z*-score. Analysis of variance was performed to compare the *Z*-scores of regional volumes of the patient at t1 and t2, with the Bonferroni *post hoc* test. To represent the changes in regional brain volumes of the patient after 3 years, we generated heat maps of *Z*-scores from t1 and t2 for each region of interest using the R software version 4.0.4 and the package *pheatmap*.

### Volumetric analysis

**Figure 1:**
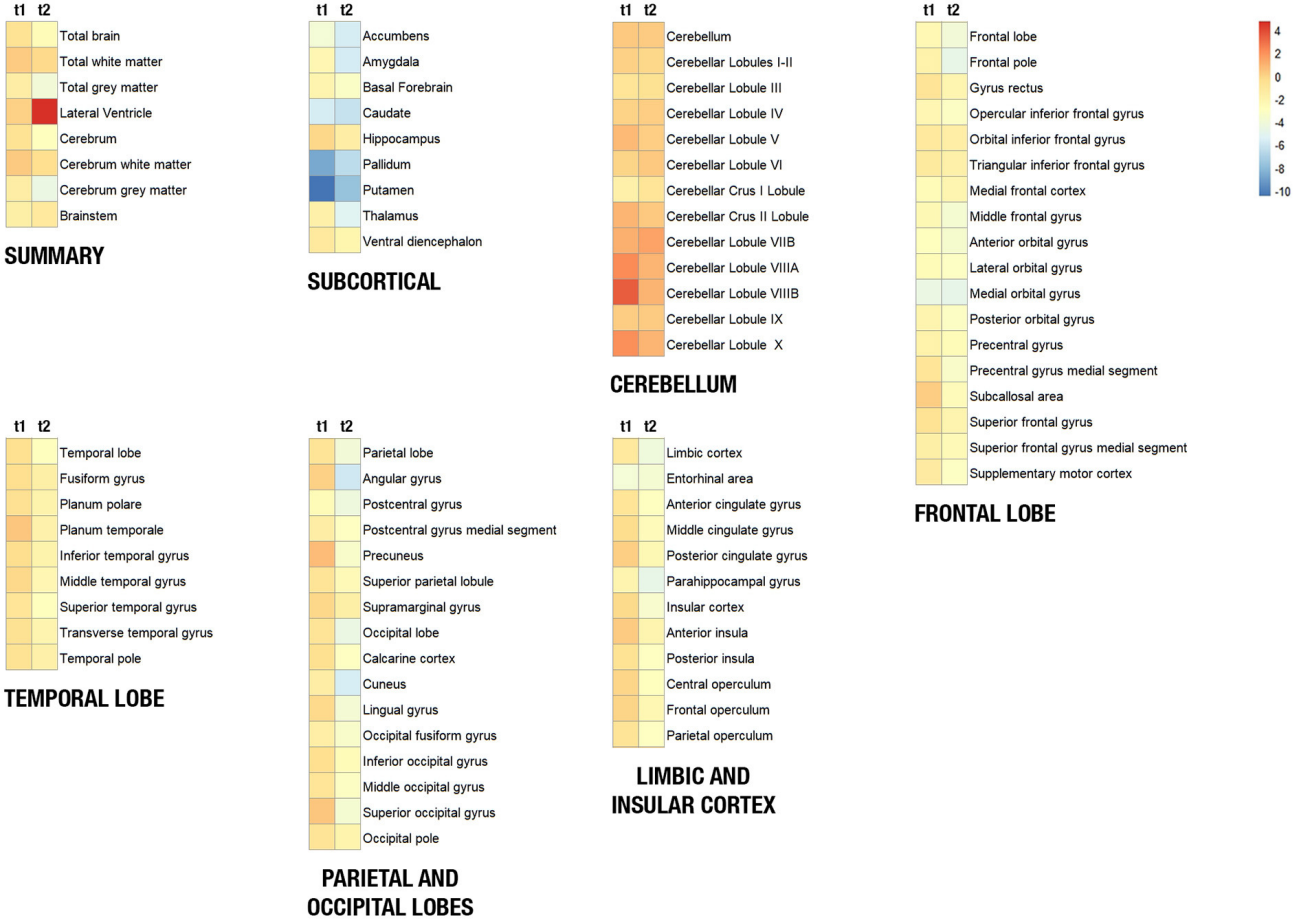
Heat maps showing the volume of brain structures of the patient with juvenile Huntington's disease at 18 years (t1) and 21 years (t2). Brain structures were clustered in seven regions (summary, frontal lobe, temporal lobe, parietoccipital lobe, limbic and insular cortex, subcortical region, and the cerebellum). The blue-to-red scale indicates lower to higher intensity levels based on a *Z*-score of normalized volumes for each structure. Red/orange squares represent that the patient's structure volume is higher than its mean volume in controls, and yellow/blue squares represent that the patient's structure volume is lower than its mean volume in controls.

**Figure 2:**
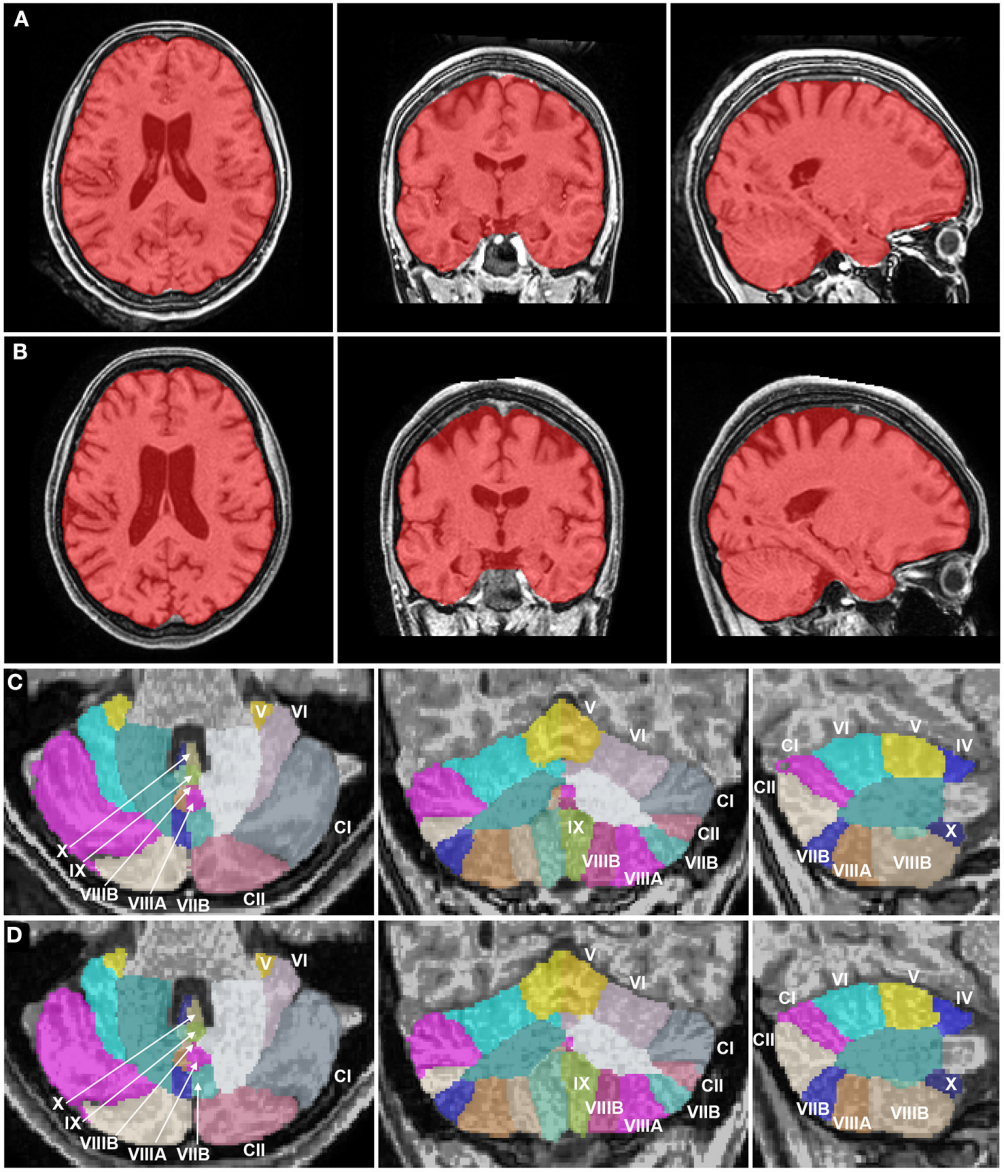
Brain magnetic resonance imaging segmentation maps of the patient with juvenile Huntington's disease at 18 years and 21 years. **(A, B)** Whole brain images (in red) of the patient in axial, coronal, and sagittal planes show an atrophy of the striatum at 18 years **(A)**, progressing to a more diffuse process of atrophy, including subcortical structures and neocortical areas like the frontal and parietal lobes at 21 years **(B)**. White matter volume was relatively preserved as compared to gray matter. **(C, D)** Cerebellum images of the patient in axial, coronal, and sagittal planes show a normal cerebellar volume and a volumetric stability at 18 years **(C)** and 21 years **(D)**. The Roman numerals of the cerebellar lobules were added. CI, Crus I; CII: Crus II.

The volumes of the brain regions of the patient with adolescence-onset HD and normal controls were compared (Table 1). At 18 years of age (t1), the subcortical region was the most affected by atrophy compared to other regions (*F*_(6, 77)_ = 9.99, *p* < 0.001). At 21 years of age, there was a more diffuse process of atrophy. The cerebellum was spared from atrophy in both t1 and t2 (Figure 1). At 18 years of age (t1), the *Z*-score of cerebellar volume of the patient was higher than subcortical structures and frontal lobe (cerebellum vs. subcortical region: Bonferroni mean difference = 4.67, *p* < 0.001; cerebellum vs. frontal lobe: Bonferroni mean difference = 2.45, *p* < 0.001). At 21 years of age (t2), the *Z*-score of cerebellar volume of the patient was higher than all other regions (*F*_(6, 77)_ = 22.76, *p* < 0.001; cerebellum vs. subcortical region: Bonferroni mean difference = 5.21, *p* < 0.001; cerebellum vs. parietoocciptal lobe = 3.87, *p* < 0.001; cerebellum vs. limbic and insular cortex: Bonferroni mean difference = 3.45, *p* < 0.001 cerebellum vs. frontal lobe: Bonferroni mean difference = 3.34, *p* < 0.001; cerebellum vs. temporal lobe: Bonferroni mean difference = 2.46, *p* < 0.001). There was no marked difference in the volume of distinct lobules of cerebellum in t1 and t2 (Figure 2). There was not difference in brain region volumes between control group 1 and 2.

**Table 1:** Volumes of brain structures in the patient with juvenile Huntington's disease and controls.

**Brain structure**	**18 years old (t1)**			**21 years old (t2)**			
	**Patient** **at t1**	**Control group 1 (*****n*** = **8), mean**	***Z*** **-score**	**Patient at t2**	**Control group 2 (*****n*** = **11), mean**	***Z*** **-score**	**% Volume change from t1 to t2**
Intracranial volume	1,249.23	1,343.53	−0.72	1,156.04	1,332.83	−1.93	−7.46
Cerebrum total	969.59	1032.80	−0.65	833.39	1042.04	−2.88	−14.05
Cerebrum cortex	526.39	603.16	−1.57	399.94	595.18	−4.51	−24.02
Cerebrum white	443.20	429.64	0.27	433.44	446.86	−0.40	−2.20
Caudate	1.56	7.60	−5.74	1.20	7.68	−6.27	−22.82
Putamen	3.11	8.90	−10.53	2.51	8.50	−7.75	−19.26
Pallidum	1.04	2.95	−8.73	1.42	2.87	−6.55	36.99
Thalamus	9.69	12.23	−1.77	6.37	12.39	−5.23	−34.30
Frontal lobe	156.89	192.76	−2.39	127.03	183.96	−4.01	−19.03
Temporal lobe	111.66	117.54	−0.50	88.82	116.13	−2.90	−20.46
Parietal lobe	98.26	104.46	−0.75	70.79	107.06	−3.99	−27.96
Occipital lobe	66.92	72.68	−0.72	42.83	73.85	−4.49	−36.00
Insular cortex	29.63	30.25	−0.24	21.66	30.60	−3.57	−26.90
Limbic cortex	37.75	42.39	−1.18	28.45	40.87	−4.17	−24.64
Cerebellum total	128.17	126.14	0.31	119.63	117.11	0.23	−8.54
Cerebellar lobule I–II	0.11	0.11	0.01	0.11	0.12	−0.12	0.00
Cerebellar lobule III	1.33	1.47	−0.82	1.35	1.50	−0.61	0.02
Cerebellar lobule IV	4.31	4.32	−0.02	4.23	4.14	0.14	−0.08
Cerebellar lobule V	8.37	7.74	0.78	7.86	7.75	0.15	−0.51
Cerebellar lobule VI	17.50	17.66	−0.07	16.21	15.53	0.31	−1.29
Cerebellar crus I lobule	22.80	28.35	−1.71	20.88	24.22	−0.81	−1.92
Cerebellar crus II lobule	17.61	16.25	0.90	16.35	15.66	0.32	−1.26
Cerebellar lobule VIIB	10.48	9.22	1.06	10.36	9.39	1.41	−0.12
Cerebellar lobule VIIIA	13.64	11.26	2.19	11.87	10.23	0.92	−1.77
Cerebellar lobule VIIIB	9.64	7.29	3.42	8.73	7.40	0.95	−0.91
Cerebellar lobule IX	7.02	6.82	0.16	6.89	6.54	0.23	−0.13
Cerebellar lobule X	1.34	1.11	2.02	1.18	1.01	0.91	−0.16

Values are presented in cm^3^. Volume change from t1 to t2 is presented in percentage.

## Discussion

The present case report described a patient with adolescence-onset HD presenting Parkinsonism without hyperkinetic movements or ataxic abnormalities. There was no cerebellar volume reduction in a patient with JHD compared to other brain regions, and this relative sparing of the cerebellum was still evident after 3 years. Also, the cerebellar volume of the patient was similar to sex- and age-matched controls. These results corroborate with former findings indicating that the cerebellum may be preserved or enlarged in cases of adolescence-onset HD due to a compensatory mechanism (8).

According to the neurodevelopmental theory of neurodegeneration, early compensatory mechanisms may arise in the developing brain affected by a neurodegenerative process, allowing normal function in childhood (13). A recent study showed that children with expanded CAG repeats present a hyperconnectivity between the striatum and the cerebellum decades before the onset of HD. The connectivity of the cerebellar-striatal circuitry was not altered in children without expanded CAG repeats. The input from the subthalamic nucleus to the cerebellum was affected by the number of CAG repeats (14). These findings support the compensatory role of the cerebellum in HD.

The interaction between the basal ganglia and the cerebellum circuitries has a role in many physiological functions, such as reward/motivation, sensorimotor adaptation, and learning (15). In the early stages of adult-onset HD, there is a preferential degeneration of striatal medium-sized spiny neurons associated with the indirect pathway, leading to a dysfunctional indirect pathway which results in chorea (16). Evidence shows that the integration between the basal ganglia and the cerebellum occurs through the indirect pathway, *via* subthalamic nucleus—pontine nuclei—dentate nucleus (15).

Considering the evidence of cerebellar sparing in adolescence-onset HD and the development of a hyperconnectivity between the basal ganglia and the cerebellum in pre-manifest children with expanded CAG repeats, it is reasonable to suppose that the cerebellum may modulate the dysfunctional indirect pathway in adolescence-onset HD, postponing the onset of hyperkinetic movements. This compensatory mechanism depends on the integrity of the cerebellum, as seen in our patient. The concomitant degeneration of the direct pathway caused by HD neurodegeneration and the compensatory cerebellar-striatal hyperconnectivity may lead to the parkinsonian symptoms.

Despite having no direct correlation between the number of CAG expansions and cerebellar atrophy (3, 17), the rate of cerebellar volume reduction may be more accelerated in patients with high CAG repeats (18). The number of CAG repeats may also determine the existence of a compensatory mechanism in cerebellar-striatal circuitry. In adolescence-onset HD, a moderate-to-high number of CAG repeats (50–100 copies) may cause critical neurodegeneration in vulnerable regions, such as the striatum but sparing the cerebellum. Otherwise, in extreme CAG repeats (>100 copies), the neurodegenerative process is globally severe, without regional sparing, as seen in childhood-onset HD.

To illustrate this dissociation between age at onset of HD and cerebellar atrophy, a previous neuropathological study showed that a 6-year-old boy with childhood-onset HD and 169 CAG repeats presented several microscopic abnormalities in the cerebellar cortex, as marked loss of Purkinje cells and thinning of the molecular layer, as well as the presence of huntingtin-immunopositive intranuclear inclusions in remaining Purkinje cells (19). The same study described a relatively intact cerebellum of his father, who had adult-onset HD (54 CAG repeats). Unfortunately, there are no neuropathological studies with adolescence-onset HD.

As a case report, our study was limited by the sample size. Also, we did not perform genetic testing on the patient's father and other relatives. The control group subjects were not longitudinally the same in t1 and t2. MRI 1.5 T images may not have contributed to a more accurate brain volumetry, and few studies report volumetric data of the cerebellum using the vol2Brain/CERES automatic segmentation methodology.

Our results support that the cerebellum may have a critical role in HD, particularly in juvenile forms of the disease, but it is still not possible to draw conclusions of this hypothesized mechanism with only one case. Further studies must explore the cerebellar sparing in adolescence-onset HD with larger samples and longitudinal follow-up of patients.

## Data availability statement

The raw data supporting the conclusions of this article will be made available by the authors, without undue reservation.

## Ethics statement

Ethical review and approval was not required for the study on human participants in accordance with the local legislation and institutional requirements. The patients/participants provided their written informed consent to participate in this study.

## Author contributions

JR and LR carried out the patient information acquisition. BS-L and LR performed the volumetric analysis. BS-L drafted the manuscript and prepared the figures and table. All authors revised and approved the final manuscript.
